# Comparative safety, efficacy, and predictors of complete occlusion of flow diverter devices in the treatment of unruptured distal anterior cerebral artery aneurysms

**DOI:** 10.1007/s10072-026-08869-w

**Published:** 2026-02-18

**Authors:** Hamza Adel Salim, Luca Scarcia, Frédéric Clarençon, Orabi Hajjeh, Motaz Daraghma, Davide Simonato, Yan-Lin Li, Eimad Shotar, Kevin Premat, Pascal Jabbour, Stavropoula I. Tjoumakaris, Reid M. Gooch, Marios Psychogios, Nikos Ntoulias, Peter Sporns, Ajit S. Puri, Jasmeet Singh, Anna Luisa Kuhn, Ameer E. Hassan, Oktay Algin, Markus A. Möhlenbruch, Sophia Hohenstatt, Riccardo Russo, Mauro Bergui, Oded Goren, Matthew J. Kole, Nourou Dine Adeniran Bankole, Richard Bibi, Gregoire Boulouis, Takeshi Morimoto, Fumihiro Sakakibara, Raoul Pop, Ciprian Juravle, Joanna W. K. Ho, Ãngel Ferrario, Virginia Pujol Lereis, Jared Cooper, Chirag D. Gandhi, Giancarlo Salsano, Lucio Castellan, Arturo Consoli, Alessandro Sgreccia, Eytan Raz, Charlotte Chung, Julien Burel, Chrysanthi Papagiannaki, Umair Rasheed, Khawaja Muhammad Baqir Hassan, Hong Tao, Zhe Ji, Riitta Rautio, Matias Sinisalo, Maria Ruggiero, Elvis Lafe, Valerio Da Ros, Luigi Bellini, Joseph Domenico Gabrieli, Francesco Causin, Michael R. Levitt, Kate Carroll, Zachary Abecassis, Antonio Armando Caragliano, Sergio Lucio Vinci, Guillaume Bellanger, Christophe Cognard, Gaultier Marnat, Lisa Saleille, Nicola Limbucci, Francesco Capasso, Mariangela Piano, Claudia Rollo, Alexis Guedon, Francesco Arpaia, Andrea Romi, Fortunato Di Caterino, Alessandra Biondi, Erwah Kalsoum, Mykola Vyval, Adrien Guenego, Than Nguyen, Mohamad Abdalkader, Thibault Agripnidis, Aman B. Patel, Vitor Mendes Pereira, Maurizio Fuschi, Alessandro Pedicelli, Vivek Yedavalli, Max Wintermark, Andrea M. Alexandre, Adam A. Dmytriw

**Affiliations:** 1 Department of Neuroradiology, MD Anderson Medical Center, 1515 Holcombe Blvd, Unit 1473, Houston, TX 77030 USA; 2 Department of Radiology, Division of Neuroradiology, Johns Hopkins Medical Center, Baltimore, MD USA; 3 Neuroradiology Unit, Henri Mondor Hospital, Creteil, France; 4 Department of Neuroradiology, Pitié-Salpêtrière Hospital, Sorbonne University, Bd de L’Hôpital, APHP, 47, Paris, France; 5 Sorbonne University, Paris, France; 6 Department of Neuroradiology, Oxford University Hospitals NHS Foundation Trust, Nuffield, Oxford, UK; 7 Departments of Clinical Neurosciences and Surgical Sciences, University of Oxford, Oxford, UK; 8 Department of Neurosurgery, Thomas Jefferson University Hospital, Philadelphia, PA USA; 9 Diagnostic and Interventional Neuroradiology, Universitätsspital Basel, Basel, Switzerland; 10 Department of Radiology and Neuroradiology, Stadtspital, Zürich, Switzerland; 11 Division of Neurointerventional Radiology, University of Massachusetts Medical Center, Worcester, MA USA; 12 Department of Neurology, Valley Baptist Medical Center, University of Texas Rio Grande Valley, Harlingen, TX USA; 13 Department of Radiology, Ankara University, Ankara, Turkey; 14 Interventional Neuroradiology, Heidelberg University Hospital, Heidelberg, Germany; 15 Department of Neuroscience, Neuroradiological Unit, University of Turin, Azienda Ospedaliera Città Della Salute E Della Scienza Hospital, Turin, Italy; 16 Department of Neurosurgery, Geisinger Health System, Danville, PA USA; 17 Clinical Investigation Center (CIC), 1415, INSERM, Teaching Hospital of Tours, Tours, France; 18 Department of Data Science, Hyogo Medical University, Nishinomiya, Japan; 19 Interventional Neuroradiology Department, Strasbourg University Hospitals, Strasbourg, France; 20 Department of Neurosurgery, Kwong Wah Hospital, Hong Kong, China; 21 Servicio de Neurorradiologa Intervencionista, Fleni, Buenos Aires, Argentina; 22 Department of Neurology, Vascular Neurology Division, Institute of Neurological Research, FLENI, Buenos Aires, Argentina; 23 Department of Neurosurgery, Westchester Medical Center, New York, USA; 24 UO Neuroradiologia, IRCCS Ospedale Policlinico San Martino, Genoa, Italy; 25 Diagnostic and Interventional Neuroradiology Department, Foch Hospital, Suresnes, France; 26 University of Versailles, Versailles, Saint Quentin-Des-Yvelines France; 27 Department of Radiology and Neurosurgery, NYU Langone Health, New York, NY USA; 28 Department of Radiology, Rouen University Hospital, Rouen, France; 29 Department of Neuroradiology, Lahore General Hospital, Lahore, Pakistan; 30 Department of Neurosurgery, Xuanwu Hospital, Capital Medical University, Beijing, China; 31 Department of Interventional Radiology, Turku University Hospital, Turku, Finland; 32 Neuroradiology Unit, AUSL Romagna, Cesena, Italy; 33 Department of Biomedicine and Prevention, University Hospital of Rome Tor Vergata, Rome, Italy; 34 Neuroradiology Unit, Policlinico Universitario Di Padova, Padua, Italy; 35 Department of Neurological Surgery, University of Washington, Seattle, WA USA; 36 Neuroradiology Unit, AOU Policlinico G. Martino, Messina, Italy; 37 Service de Neuroradiologie Interventionnelle Et Diagnostique, Hôpital Purpan CHU Toulouse, Toulouse, France; 38 Neuroradiology Department, Bordeaux University Hospital, Bordeaux, France; 39 Interventional Neurovascular Unit, A.O.U. Careggi, Florence, Italy; 40 Neuroradiology Unit, ASST Grande Ospedale Metropolitano Niguarda, Milan, Italy; 41 Department of Neuroradiology, AP-HP Nord, Hôpital Lariboisière, Paris, France; 42 Neuroradiology Unit, IRCCS Policlinico San Matteo, Pavia, Italy; 43 Department of Interventional Neuroradiology, University Hospital Centre Besancon, Besancon, France; 44 Research and Practical Centre for Endovascular Neuroradiology, NAMS of Ukraine, Kiev, Ukraine; 45 Department of Diagnostic and Interventional Neuroradiology, Erasme University Hospital, Brussels, Belgium; 46 Department of Radiology and Neurology, Boston University Chobanian and Avedisian School of Medicine, Boston, MA USA; 47 Neuroradiology Department, University Hospital Timone, AP-HM, 13005 Marseille, France; 48 Neuroendovascular Program, Massachusetts General Hospital & Brigham and Women’s Hospital, Harvard University, Boston, MA USA; 49 Neurovascular Centre & RADIS Lab, St. Michael’s Hospital, University of Torontoand, Toronto Metropolitan University, Toronto, Canada; 50 UOSA Neuroradiologia Interventistica, Fondazione Policlinico Universitario A.Gemelli IRCCS, Rome, Italy

**Keywords:** Flow diverter devices, Distal anterior cerebral artery aneurysms, Endovascular treatment, Aneurysm occlusion

## Abstract

**Background:**

Flow diverters (FDs) are increasingly used for cerebral aneurysms, including distal anterior cerebral artery (DACA) aneurysms, but comparative data between devices in this challenging location are limited.

**Objective:**

To compare the safety and efficacy of Pipeline, Silk Vista Baby (SVB), and FRED Jr. FDs for unruptured DACA aneurysms and identify predictors of complete occlusion.

**Methods:**

We retrospectively analyzed 166 patients treated with FDs at 39 centers in 14 countries (2018–2022) from the CRETA registry. Outcomes included aneurysm occlusion (O’Kelly–Marotta [OKM] scale), complications, retreatment, modified Rankin Scale (mRS) scores, and independent predictors of complete occlusion using multivariable Cox regression.

**Results:**

Aneurysms were predominantly saccular and located on the pericallosal artery. Complete occlusion (OKM D) was achieved in 73%, and neck remnants (OKM C) in 12%, with no differences across devices. Ischemic complications occurred in 11% (mostly asymptomatic), hemorrhagic complications in 5%, and in-stent stenosis in 17%. Retreatment was performed in 1.3%. At last follow-up, 98% had mRS ≤ 2. Independent predictors of complete occlusion were female sex (HR 1.85), asymptomatic presentation (HR 1.79), smaller aneurysm neck (HR 0.83/mm), radial access (HR 2.20), and aspirin plus ticagrelor therapy (HR 1.84); device type was not predictive.

**Conclusion:**

Pipeline, SVB, and FRED Jr. FDs show similar safety and efficacy for unruptured DACA aneurysms. Complete occlusion is influenced by clinical and procedural factors, supporting individualized device selection.

## Introduction

Flow diverting stents (FDs) have been a major technological advance in cerebral aneurysm treatment. [1, 2] Commonly used devices are Pipeline (Medtronic), Silk Vista Baby (SVB) [Balt Extrusion], Flow Redirection Endoluminal Device (FRED), and Surpass Evolve (Stryker). [3–5] These devices induce gradual thrombosis and vessel remodeling by rerouting blood flow along the parent artery and away from the aneurysm sac due to endothelialization around the aneurysm neck over time, leading to the exclusion of the aneurysm from the vasculature. [5, 6] Despite favorable results reported for individual FDs, few studies directly compare the safety and efficacy of FDs in different cerebrovascular territories. [7, 8]

Distal anterior cerebral artery (DACA) aneurysms are particularly challenging because of their anatomical characteristics. [9] These aneurysms are relatively rare, representing about 5–6% of all intracranial aneurysms, and are difficult to access because of their anatomical peculiarities. [9] Notably, a majority of these aneurysms rupture while they are still small (< 5 mm) and prior to developing mass effect, frequently presenting with subarachnoid hemorrhage or intracerebral hematoma**.** Surgical treatment is complicated due to factors such as the inability to achieve proximal control, narrow working space, and tight adhesion of the aneurysm to surrounding tissues. [9, 10] Due to the small caliber of parent vessels and complex branch morphology, FDs deployment in this area can be technically challenging [11, 12]. Despite the increasing use of FDs, the safety and efficacy of these devices in distal territories are debated [11, 13]. FDs across the anterior cerebral artery may have functional and hemodynamic implications given its role in executive function, behavior, and emotional regulation [14]. FDs offer both the feasibility and effectiveness of treatment of DACA aneurysms, with high rates of aneurysm occlusion but with a contaminant risk of both ischemic and hemorrhagic complications. [11]

Currently, devices are used based on device availability in the institution and according to the preference of the neurointerventionalist, given the limited data on the performance of different FD types for unruptured DACA aneurysms. [7, 12]

Beyond device-related outcomes, predictors of successful aneurysm occlusion following flow diversion in DACA remain poorly defined. Identifying clinical, anatomical, and procedural factors associated with complete occlusion may help optimize patient selection, procedural strategy, and post-procedural management. Accordingly, this study aimed to compare the safety and efficacy of commonly used flow diverters for unruptured DACA aneurysms and to identify independent predictors of complete aneurysm occlusion.

## Methods

### Patients and study design

Data in the Clinical and Radiological Evaluation of endovascular Treatment of distal anterior cerebral artery Aneurysms (CRETA) registry were collected from 39 sites in 14 countries (Argentina, Austria, Belgium, China, Finland, France, Germany, Italy, Japan, Pakistan, Switzerland, Turkey, Ukraine, and USA). [15] A prior analysis of this same CRETA Registry cohort was recently published by Scarcia et al. [15] Unlike that report, which provided a general evaluation of safety and efficacy, the present study focuses specifically on device-level comparisons among flow diverters used to treat unruptured DACA aneurysms as well as on identifying predictors of complete occlusion. The study followed the Strengthening the Report of Observational Studies in Epidemiology (STROBE) reporting guidelines.

Participating centers retrospectively abstracted data from consecutively admitted patients for endovascular treatment of DACA aneurysms, defined as those located in the anterior cerebral artery, distal to the anterior communicating artery. Patients with aneurysms involving the anterior communicating artery or the A1-A2 junction were excluded. In the current analysis we considered only patients treated with FDs for unruptured DACA aneurysms. We included patients aged ≥ 18 years treated between January 2018 to December 2022. Data were self-reported by each center and homogenized by local neurointerventionalists prior to submission. Data variables included demographics (age and sex), clinical history (hypertension, smoking, family history of aneurysm), neurologic evaluation on admission (headache, focal neurological deficit), radiographic features of the aneurysm (morphology, maximum size, neck width, branch involvement, parent vessel size), procedural details, medications, complications, and radiological and clinical outcomes at discharge and at most recent follow-up.

### Medications and procedure technique

All patients were treated under general anesthesia via a transfemoral or transradial approach with systemic heparinization using a coaxial or triaxial system. Each FDs (manufacturer, model, size, and number implanted) were chosen according to the preference of the neurointerventionalist. The indication for treatment, antiplatelet therapy regimen, duration, and decision to perform pre-procedural platelet function testing as well as the anticoagulation protocol was based on the institution’s standard of care. Platelet function testing was performed in 89 of 166 patients (57%), primarily using light transmission aggregometry. Clopidogrel resistance was observed in 19.8% of tested patients, who were switched to ticagrelor.

### Outcomes

The primary outcome was complete occlusion of the target aneurysm at the last radiographic follow-up using the O’Kelly–Marotta classification (OKM), [16] where OKM A is defined as complete/near-complete aneurysm filling (> 95%), B as incomplete aneurysm filling (5–95%), C as neck remnant (< 5%), and D as complete occlusion. Procedural outcomes included rates of adequate aneurysm occlusion (OKM C-D), technical success, rates of retreatment, incidence and type of complications, radiological outcome of the covered branches, mortality, and modified Rankin Scale (mRS) score. Follow-up imaging was primarily performed using digital subtraction angiography (DSA). In cases where DSA was not available or feasible, magnetic resonance angiography (MRA) or computed tomography angiography (CTA) was used instead. Patients whose occlusion status was assessed solely using the Raymond- Roy (RR) classification were translated to the OKM scale for the conclusive analysis. In this conversion, RR1 was classified as equivalent to OKM D, RR2 to OKM C, and RR3 to OKM B and A. In-stent stenosis was defined as narrowing of at least 50% of the vessel lumen at radiological follow-up.

### Statistical analysis

Categorical variables were summarized as frequencies and percentages and compared using Fisher’s exact test, given that some subgroups contained fewer than five observations. Continuous variables were summarized as medians with interquartile ranges (IQR) and compared using the Kruskal–Wallis test for multiple-group comparisons.

Time-to-complete aneurysm occlusion was analyzed using Cox proportional hazards regression. Univariable Cox models were used to assess associations between clinical, anatomical, and procedural variables and complete occlusion. Variables with P value < 0.1 in univariable analyses were entered into a multivariable Cox model. Hazard ratios (HRs) with 95% confidence intervals (CIs) were reported. In addition, Kaplan–Meier curves stratified by flow-diverter device were generated to visualize time to complete occlusion, and groups were compared using the log-rank test.

A two-sided p-value < 0.05 was considered statistically significant. R statistical software (version 4.3.0, R Project for Statistical Computing) and Rstudio statistical software (version 2023.03.0 + 386, Rstudio) were used for statistical analyses and data visualization.

## Results

### Patient characteristics

A total of 166 patients met the inclusion criteria. Among these, 76 patients were treated with Silk Vista Baby, 59 with Pipeline, 22 with FRED Jr., and 9 with other flow-diverting devices (Surpass, Derivo, and P48). Median age was 61 years (IQR, 52–67); 74% were women. Hypertension (57%), smoking (21%), and hyperlipidemia (28%) were common comorbidities. Patient characteristics were well balanced among groups treated with SVB, FRED Jr, Pipeline, or other FD types, with no statistically significant differences. (Table 1).

**Table 1:** Baseline clinical, anatomical, and procedural characteristics of patients with unruptured DACA treated with flow diversion

Variable	Overall	Silk Vista Baby	Fred Jr	Other types (Surpass, Derivo and P48)	Pipeline	P^*2*^
	N = 166^*1*^	N = 76^*1*^	N = 22^*1*^	N = 9^*1*^	N = 59^*1*^	
Sex						0.7
Male	40 (26%)	18 (27%)	7 (33%)	1 (11%)	14 (24%)	
Female	116 (74%)	49 (73%)	14 (67%)	8 (89%)	45 (76%)	
Age (years)	61 (52, 67)	61 (53, 67)	62 (47, 68)	63 (61, 68)	61 (53, 67)	0.6
Smoking	34 (21%)	19 (26%)	4 (19%)	3 (33%)	8 (14%)	0.3
Race						0.4
White	132 (80%)	60 (79%)	18 (82%)	8 (89%)	46 (78%)	
Black	23 (14%)	13 (17%)	3 (14%)	0 (0%)	7 (12%)	
Hispanic	4 (2.4%)	2 (2.6%)	0 (0%)	1 (11%)	1 (1.7%)	
Other	7 (4.2%)	1 (1.3%)	1 (4.5%)	0 (0%)	5 (8.5%)	
Hypertension	94 (57%)	46 (61%)	9 (41%)	7 (78%)	32 (54%)	0.2
Hyperlipidemia	47 (28%)	19 (25%)	4 (19%)	4 (44%)	20 (34%)	0.3
Diabetes	22 (13%)	12 (16%)	3 (14%)	1 (11%)	6 (10%)	0.9
Coronary Disease	9 (5.6%)	4 (5.6%)	1 (4.5%)	1 (11%)	3 (5.1%)	0.8
Prior Stroke	15 (9.1%)	6 (8.0%)	3 (14%)	0 (0%)	6 (10%)	0.7
Family History of Aneurysm						0.3
No	137 (85%)	59 (82%)	18 (86%)	8 (89%)	52 (88%)	
Yes, unruptured	11 (6.8%)	5 (6.9%)	3 (14%)	1 (11%)	2 (3.4%)	
Yes, ruptured	13 (8.1%)	8 (11%)	0 (0%)	0 (0%)	5 (8.5%)	
Baseline mRS 0–2	163 (99%)	74 (100%)	21 (95%)	9 (100%)	59 (100%)	0.2
Previous SAH						0.074
No	131 (79%)	55 (72%)	15 (68%)	9 (100%)	52 (90%)	
Yes, due to index aneurysm	27 (16%)	17 (22%)	6 (27%)	0 (0%)	4 (6.9%)	
Yes, due to other aneurysm	7 (4.2%)	4 (5.3%)	1 (4.5%)	0 (0%)	2 (3.4%)	

^*1*^ n (%); Median (Q1, Q3)

^*2*^ Fisher’s exact test; Kruskal–Wallis rank sum test

### Aneurysm and procedural characteristics

Aneurysms were predominantly saccular (87%) and commonly located on the pericallosal artery (60%). Median aneurysm width was 4.25 mm (IQR, 3–5.70 mm), median neck size 3 mm (IQR, 2.20–4 mm), and parent artery diameter 1.93 mm (IQR, 1.70–2.23 mm). Pipeline had fewer daughter sacs compared to SVB (22% vs. 42%; P = 0.011). Despite a higher prevalence of daughter sacs in the SVB group, this anatomical feature was not associated with differences in outcomes and likely reflects baseline case heterogeneity. Most procedures involved single-FD implantation (median, 1 device; IQR, 1–1). Dual antiplatelet therapy predominantly aspirin plus clopidogrel (77%). Procedure length was significantly shorter with SVB and FRED Jr compared to other types (median, 75 vs. 124 min; P = 0.018). (Table 2).

**Table 2:** Clinical, anatomical, and procedural characteristics of unruptured distal anterior cerebral artery aneurysms treated with flow diversion

Variable	Overall	Silk Vista Baby	Fred Jr	Other types (Surpass, Derivo and P48)	Pipeline	P^*2*^
	N = 166^*1*^	N = 76^*1*^	N = 22^*1*^	N = 9^*1*^	N = 59^*1*^	
Number of Aneurysms	1 (1, 2)	1 (1, 1)	1 (1, 2.50)	2 (1, 3)	1 (1, 2)	.008
Artery Involved						
ACA A2	38 (23%)	10 (13%)	7 (33%)	3 (33%)	18 (31%)	
ACA Pericallosal	99 (60%)	59 (78%)	9 (43%)	6 (67%)	25 (42%)	
ACA A3	25 (15%)	7 (9.2%)	4 (19%)	0 (0%)	14 (24%)	
ACA A4	3 (1.8%)	0 (0%)	1 (4.8%)	0 (0%)	2 (3.4%)	
ACA Type						0.3
ACA Paired	129 (81%)	60 (83%)	13 (65%)	8 (89%)	48 (83%)	
ACA Azygos	10 (6.3%)	4 (5.6%)	1 (5.0%)	0 (0%)	5 (8.6%)	
ACA Biemispheric	20 (13%)	8 (11%)	6 (30%)	1 (11%)	5 (8.6%)	
Side						0.7
Right	69 (42%)	29 (38%)	11 (50%)	4 (44%)	25 (42%)	
Left	84 (51%)	43 (57%)	9 (41%)	4 (44%)	28 (47%)	
Midline	13 (7.8%)	4 (5.3%)	2 (9.1%)	1 (11%)	6 (10%)	
Morphology						0.8
Saccular	145 (87%)	68 (89%)	18 (82%)	9 (100%)	50 (85%)	
Fusiform	14 (8.4%)	6 (7.9%)	2 (9.1%)	0 (0%)	6 (10%)	
Dissecting	7 (4.2%)	2 (2.6%)	2 (9.1%)	0 (0%)	3 (5.1%)	
Aneurysm Width (mm)	4.25 (3, 5.70)	4 (3.05, 5.50)	4.70 (3, 7.20)	5 (4, 5.70)	4.10 (2.70, 5.70)	0.6
Aneurysm Depth (mm)	3.71 (2.60, 5)	3.70 (2.40, 5)	3.45 (2.80, 7)	4 (3.80, 5)	3.70 (2.70, 5)	0.6
Aneurysm Height (mm)	4.05 (3, 6)	4 (2.80, 6)	3.65 (2.80, 5.10)	6 (5, 6)	4.15 (3, 5.30)	0.3
Aneurysm Neck (mm)	3 (2.20, 4)	3 (2.10, 4)	3 (2.20, 3.50)	4 (3, 5)	3 (2.30, 4.45)	0.3
Aspect Ratio	1.32 (1, 1.70)	1.40 (0.97, 1.90)	1.43 (0.90, 1.70)	1.30 (1.20, 2)	1.22 (1, 1.57)	0.7
Dome-to-Neck Ratio	1.24 (1, 1.66)	1.30 (1.02, 1.70)	1.30 (1.10, 1.70)	1.20 (1.03, 1.50)	1.14 (1, 1.40)	0.15
Daughter Sac	50 (30%)	32 (42%)	5 (24%)	0 (0%)	13 (22%)	0.011
Parent Artery Diameter (mm)	1.93 (1.70, 2.23)	2 (1.70, 2.10)	1.75 (1.40, 1.90)	1.80 (1.80, 2.10)	2 (1.80, 2.30)	0.057
Branch Artery Origin						0.5
from the sac	37 (25%)	20 (29%)	1 (6.7%)	2 (22%)	14 (26%)	
from the neck	63 (43%)	29 (42%)	10 (67%)	4 (44%)	20 (38%)	
from the artery	46 (32%)	20 (29%)	4 (27%)	3 (33%)	19 (36%)	
Treatment						0.3
Flow Diverter	155 (93%)	72 (95%)	21 (95%)	7 (78%)	55 (93%)	
Flow Diverter + Coiling	11 (6.6%)	4 (5.3%)	1 (4.5%)	2 (22%)	4 (6.8%)	
Number of FD	1 (1, 1)	1 (1, 1)	1 (1, 1)	1 (1, 1)	1 (1, 1)	0.2
DAPT Regimen						0.8
Aspirin + Clopidogrel	124 (77%)	58 (76%)	16 (73%)	6 (75%)	44 (79%)	
Aspirin + Prasugrel	3 (1.9%)	2 (2.6%)	1 (4.5%)	0 (0%)	0 (0%)	
Aspirin + Ticagrelor	35 (22%)	16 (21%)	5 (23%)	2 (25%)	12 (21%)	

^*1*^ n (%); Median (Q1, Q3)

^*2*^ Fisher’s exact test; Kruskal–Wallis rank sum test

### Outcomes

**Fig. 1:**
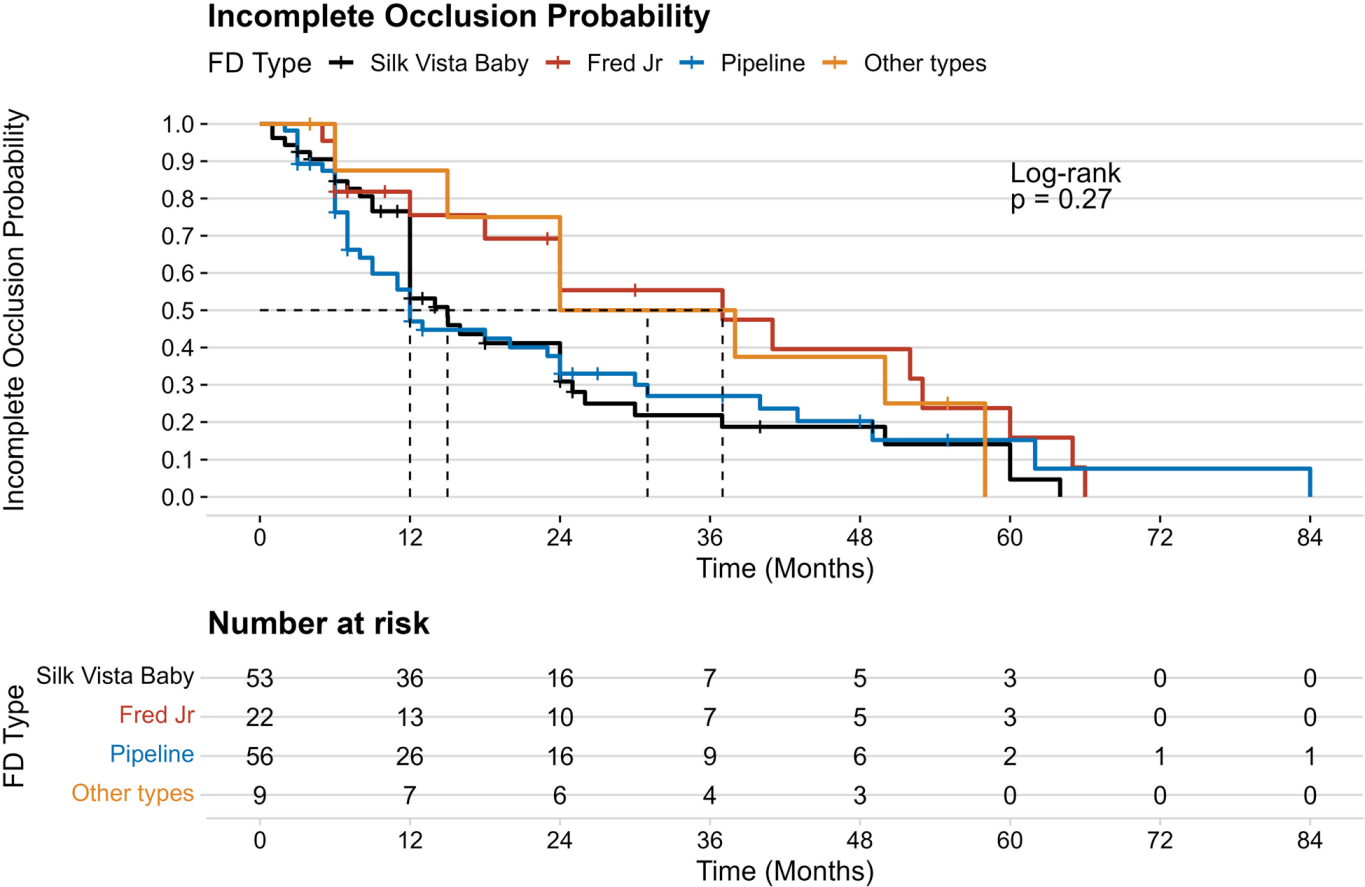
Kaplan–Meier curves for incomplete occlusion probability by flow diverter type. No significant difference in occlusion probability was observed among Silk Vista Baby, FRED Jr., Pipeline, and other devices (log-rank p = 0.27)

The median radiologic follow-up was 12 months (IQR 6–25). Immediate complete aneurysm obliteration occurred in 20% of cases, residual neck in 7.7%, and residual aneurysm in 73%, with no significant differences between FD types. Adequate occlusion at follow-up was achieved similarly across all groups. Specifically, OKM D (complete occlusion) was achieved in 73% of patients, OKM C (neck remnant) in 12%, and OKM A + B (incomplete occlusion) in 15%. Time-to-complete occlusion analysis using Kaplan–Meier curves demonstrated no significant difference in occlusion probability among device types (log-rank p = 0.27; Fig. 1). Seven patients (4.2%) were lost to radiologic follow-up. Ischemic complications occurred in 11%, mostly asymptomatic thromboembolic events (9.4%). Symptomatic ischemia with permanent neurological deficit was rare (0.6%). Hemorrhagic complications were uncommon (5%) and were predominantly intraparenchymal hemorrhage (3.3%). In-stent stenosis was observed in 17% of cases, with no significant difference among FD types. Retreatment was performed in 1.3% of patients. At baseline, 99% of patients had an mRS ≤ 2, indicating preserved functional status before treatment. At the last follow-up, 98% of patients maintained an mRS ≤ 2, reflecting overall favorable outcomes. (Table 3).

**Table 3:** Angiographic and clinical outcomes following flow diversion for unruptured distal anterior cerebral artery aneurysms

Variable	Overall	Silk Vista Baby	Fred Jr	Other types (Surpass, Derivo and P48)	Pipeline	P^*2*^
	N = 166^*1*^	N = 76^*1*^	N = 22^*1*^	N = 9^*1*^	N = 59^*1*^	
Length of Procedure (min)	80 (60, 114)	75 (58, 99)	75 (50, 112)	124 (104, 135)	81 (67, 115)	0.018
Fluoroscopy Time (min)	31 (18, 45)	35 (19, 45)	21 (15, 32)	28 (10, 38)	30 (18, 48)	0.13
Immediate RROC						0.4
Complete Obliteration	28 (20%)	12 (18%)	3 (19%)	3 (38%)	10 (19%)	
Residual Neck	11 (7.7%)	3 (4.5%)	3 (19%)	0 (0%)	5 (9.4%)	
Residual Aneurysm	104 (73%)	51 (77%)	10 (63%)	5 (63%)	38 (72%)	
RROC Last Follow-Up						0.6
Complete obliteration	116 (73%)	54 (76%)	15 (68%)	7 (78%)	40 (71%)	
Residual neck	19 (12%)	6 (8.5%)	2 (9.1%)	1 (11%)	10 (18%)	
Residual aneurysm	23 (15%)	11 (15%)	5 (23%)	1 (11%)	6 (11%)	
Access Site						0.2
No	155 (97%)	70 (99%)	19 (90%)	9 (100%)	57 (98%)	
Hematoma	2 (1.3%)	1 (1.4%)	1 (4.8%)	0 (0%)	0 (0%)	
Pseudo-aneurysm	1 (0.6%)	0 (0%)	0 (0%)	0 (0%)	1 (1.7%)	
Arterial occlusion	1 (0.6%)	0 (0%)	1 (4.8%)	0 (0%)	0 (0%)	
Ischemic Complications						0.6
No	143 (89%)	65 (90%)	18 (82%)	8 (89%)	52 (91%)	
Thromboembolic asymptomatic	15 (9.4%)	6 (8.3%)	4 (18%)	1 (11%)	4 (7.0%)	
Thromboembolic symptomatic permanent	1 (0.6%)	0 (0%)	0 (0%)	0 (0%)	1 (1.8%)	
Thromboembolic symptomatic transient	1 (0.6%)	1 (1.4%)	0 (0%)	0 (0%)	0 (0%)	
Timing of Ischemic Complication						0.3
No complication	60 (80%)	30 (81%)	6 (67%)	1 (50%)	23 (85%)	
Intra-procedural	5 (6.7%)	2 (5.4%)	2 (22%)	0 (0%)	1 (3.7%)	
Post-procedural	10 (13%)	5 (14%)	1 (11%)	1 (50%)	3 (11%)	
In-Stent Stenosis	26 (17%)	16 (23%)	3 (14%)	0 (0%)	7 (12%)	0.3
Hemorrhagic Complications						0.087
No	144 (95%)	62 (94%)	21 (100%)	6 (86%)	55 (96%)	
IPH	5 (3.3%)	4 (6.1%)	0 (0%)	1 (14%)	0 (0%)	
IVH	0 (0%)	0 (0%)	0 (0%)	0 (0%)	0 (0%)	
SAH	2 (1.3%)	0 (0%)	0 (0%)	0 (0%)	2 (3.5%)	
Vasospasm	2 (1.4%)	0 (0%)	0 (0%)	0 (0%)	2 (4.1%)	0.4
Dissection	0 (0%)	0 (0%)	0 (0%)	0 (0%)	0 (0%)	> 0.9
Air Embolization	0 (0%)	0 (0%)	0 (0%)	0 (0%)	0 (0%)	> 0.9
Deployment Issues	1 (0.6%)	0 (0%)	0 (0%)	0 (0%)	1 (1.7%)	0.6
Retreatment	2 (1.3%)	1 (1.4%)	0 (0%)	0 (0%)	1 (1.7%)	> 0.9
Last follow-up mRS 0–2	155 (98%)	69 (99%)	20 (95%)	9 (100%)	57 (98%)	0.6

^1^ n (%); Median (Q1, Q3)

^2^ Fisher’s exact test; Kruskal–Wallis rank sum test

### Predictors of complete aneurysm occlusion

In Cox proportional-hazards analyses, several variables were associated with complete occlusion in univariable models (Table 4). In the multivariable model, female sex (HR, 1.85; 95% CI, 1.09 to 3.12; P = 0.022), asymptomatic presentation (HR, 1.79; 95% CI, 1.09 to 2.94; P = 0.022), smaller aneurysm neck size (HR per millimeter increase, 0.83; 95% CI, 0.70 to 0.99; P = 0.036), radial access (HR, 2.20; 95% CI, 1.15 to 4.21; P = 0.019), and aspirin plus ticagrelor therapy (HR, 1.84; 95% CI, 1.00 to 3.39; P = 0.049) were independently associated with complete occlusion. Device type was not independently associated with complete occlusion in the multivariable model; relative to Silk Vista Baby, the HR was 0.51 (95% CI, 0.24 to 1.08; P = 0.078) for FRED Jr., 0.72 (95% CI, 0.30 to 1.75; P = 0.47) for other devices, and 0.95 (95% CI, 0.57 to 1.56; P = 0.83) for Pipeline (Table 4).

**Table 4:** Univariable and multivariable predictors of complete aneurysm occlusion following flow diversion

Characteristic	Univariable Models		MultiVariable Model	
	**HR** **(95% CI)**^*1*^	**p-value**	**HR** **(95% CI)**^*1*^	**p-value**
Sex				
Male	—		—	
Female	1.85 (1.06 to 3.24)	0.031	1.85 (1.09 to 3.12)	0.022
Age (years)	1.00 (0.99 to 1.02)	0.9		
Smoking	0.78 (0.49 to 1.25)	0.3		
Race				
White	—			
Black	1.45 (0.75 to 2.83)	0.27		
Hispanic	2.10 (0.76 to 5.79)	0.15		
Other	1.80 (0.65 to 4.98)	0.26		
Hypertension	1.10 (0.74 to 1.65)	0.63		
Hyperlipidemia	1.02 (0.66 to 1.59)	0.92		
Diabetes	1.05 (0.56 to 1.96)	0.89		
Coronary Disease	0.45 (0.11 to 1.83)	0.26		
Prior Stroke	0.92 (0.46 to 1.84)	0.82		
Family History of Aneurysm				
No	—			
Yes, unruptured	1.08 (0.50 to 2.33)	0.85		
Yes, ruptured	1.18 (0.51 to 2.72)	0.7		
Baseline mRS	0.88 (0.61 to 1.29)	0.52		
Previous SAH				
No	—			
Yes, due to index aneurysm	1.25 (0.75 to 2.10)	0.39		
Yes, due to other aneurysm	0.90 (0.28 to 2.88)	0.86		
Asymptomatic	2.28 (1.48 to 3.52)	< 0.001	1.79 (1.09 to 2.94)	0.022
Headache	0.96 (0.52 to 1.76)	0.89		
Cranial Nerve Palsy	0.30 (0.04 to 2.23)	0.24		
Weakness	0.55 (0.08 to 3.93)	0.55		
Aphasia	0.58 (0.08 to 4.17)	0.59		
Number of Aneurysms	0.94 (0.79 to 1.12)	0.5		
Artery Involved				
ACA A2	—		—	
ACA Pericallosal	0.72 (0.46 to 1.13)	0.16	0.69 (0.42 to 1.13)	0.14
ACA A3	1.11 (0.60 to 2.05)	0.73	1.40 (0.67 to 2.90)	0.37
ACA A4	0.28 (0.06 to 1.20)	0.086	0.32 (0.06 to 1.63)	0.17
ACA Type				
ACA Paired	—			
ACA Azygos	0.87 (0.40 to 1.89)	0.73		
ACA Biemispheric	0.86 (0.42 to 1.78)	0.68		
Side				
Right	—			
Left	1.00 (0.67 to 1.50)	0.99		
Midline	0.60 (0.25 to 1.40)	0.23		
Morphology				
Saccular	—			
Fusiform	1.23 (0.59 to 2.54)	0.58		
Dissecting	1.48 (0.66 to 3.33)	0.34		
Morphology				
Regular	—			
Irregular	0.81 (0.54 to 1.20)	0.29		
Aneurysm Width (mm)	0.94 (0.88 to 1.02)	0.13		
Aneurysm Depth (mm)	1.04 (0.97 to 1.12)	0.24		
Aneurysm Height (mm)	1.01 (0.98 to 1.04)	0.56		
Aneurysm Neck (mm)	0.85 (0.73 to 0.99)	0.04	0.83 (0.70 to 0.99)	0.036
Dome-to-Neck Ratio	1.04 (0.65 to 1.69)	0.86		
Daughter Sac	0.72 (0.46 to 1.12)	0.15		
Parent Artery Diameter (mm)	1.68 (1.05 to 2.69)	0.032	1.40 (0.77 to 2.53)	0.25
Branch Artery Origin				
from the sac	—			
from the neck	1.10 (0.62 to 1.96)	0.74		
from the artery	1.57 (0.88 to 2.79)	0.13		
Access Type				
Femoral	—		—	
Radial	2.09 (1.09 to 4.01)	0.026	2.20 (1.15 to 4.21)	0.019
Ulnar	3.28 (0.45 to 24.0)	0.24	0.43 (0.05 to 4.11)	0.46
Access Dimension (Fr)	1.03 (0.83 to 1.28)	0.8		
System				
Coaxial	—			
Triaxial	0.75 (0.46 to 1.23)	0.25		
Treatment				
Number of FD	0.99 (0.42 to 2.30)	0.98		
FD Type				
Silk Vista Baby	—		—	
Fred Jr	0.60 (0.33 to 1.10)	0.1	0.51 (0.24 to 1.08)	0.078
Other types (Surpass, Derivo and P48)	0.66 (0.30 to 1.49)	0.32	0.72 (0.30 to 1.75)	0.47
Pipeline	0.99 (0.63 to 1.54)	0.95	0.95 (0.57 to 1.56)	0.83
FD Diameter	1.75 (0.78 to 3.97)	0.18		
FD Length	1.01 (0.97 to 1.06)	0.65		
Length of Procedure (min)	1.00 (0.99 to 1.00)	0.11		
Fluoroscopy Time (min)	1.01 (1.00 to 1.02)	0.053	1.00 (0.99 to 1.01)	> 0.99
DAPT Regimen				
Aspirin + Clopidogrel	—		—	
Aspirin + Prasugrel	0.91 (0.13 to 6.57)	0.92	0.99 (0.12 to 8.04)	> 0.99
Aspirin + Ticagrelor	1.88 (1.16 to 3.06)	0.011	1.84 (1.00 to 3.39)	0.049

^*1*^ HR = Hazard Ratio, CI = Confidence Interval

## Discussion

This retrospective multicenter study aimed to compare the safety and efficacy of different FD devices, namely Pipeline, SVB, and FRED Jr., in the treatment of unruptured DACA aneurysms and to identify predictors of complete aneurysm occlusion. With 166 patients included from 39 centers across 14 countries, our analysis represents one of the largest device-level comparisons to date within this anatomically complex vascular territory. Our findings demonstrate high rates of aneurysm occlusion and low complication rates across all device types, suggesting that currently available FDs offer similar performance profiles in this setting. In multivariable analyses, complete occlusion was independently associated with female sex, asymptomatic presentation, smaller neck size, radial access, and aspirin ticagrelor therapy, whereas device type was not.

In our cohort, OKM C-D was achieved in the majority of patients in all device groups. These results are consistent with previously published occlusion rates after treatment of DACAs with FDs. A meta-analysis of 27 studies determined a general long-term adequate occlusion rate of 83% over a cohort of 484 distal aneurysms, supporting the wider feasibility of FDs within distal vascular territories. [17] Our results are also corroborated by device-specific series. Although originally designed for proximal vasculature, the Pipeline has demonstrated 90.2% complete occlusion and 98% adequate occlusion at 12 months in distal locations. [18] SVB, also a low-profile device for 1.5–3.5 mm vessels, also yielded near/complete occlusion at one-year follow-up in a 50-aneurysm series. [19] FRED Jr. showed complete occlusion in 100% of patients with follow-up imaging at 12 months. [20] These comparable results between devices in our study reinforce the idea that modern FDs, despite the differences in design or composition, can achieve similar efficacy.

The overall rate of procedural complications was low to moderate in our study, similar to previous reports of 10–15%, with permanent morbidity around 5%. [17] Notably, our study did not show a statistically significant difference in complication rates based on FD type, which aligns with previous reports. [21] In a large series of SVB cases, the procedural complication rate was 8%, with a neurologic morbidity rate of 4% and no deaths. [16] A study of FRED Jr. found a 7% rate of ischemic events and no mortality. [20] For Pipeline-treated distal aneurysms, 11% suffered thromboembolic events and 3.8% suffered hemorrhagic complications, whereas procedure-related mortality was approximately 5%. [18] These data support our findings that most procedural complications are asymptomatic or transient, while permanent deficiencies are rare. Moreover, although the Surpass/Derivo/P48 subgroup had the longest procedure times, this finding is difficult to generalize because the subgroup was small; thus, the observed difference may represent sampling variability rather than a consistent tendency toward longer procedures with these devices.

The clinical outcomes were favorable throughout our cohort, 98% of patients achieved a favorable outcome with mRS ≤ 2 at last follow-up and no intergroup differences. This aligns with previous reports in which favorable functional outcome (mRS 0–2) was achieved in 90–99% of patients after FDs for distal aneurysms. [18, 21] Cagnazzo et al. described permanent neurologic morbidity in 5% of treated patients, [17] and Senol et al. found an mRS ≤ 2 in 98.8% of patients with M1 aneurysms treated with FDs. [21] These outcomes support that FDs in the distal anterior circulation are not only technically feasible but well-tolerated with low risk of significant functional deterioration.

Beyond device-level comparisons, this study identified several clinical and procedural variables as possible independent predictors of complete occlusion. Smaller aneurysm neck size was associated with a higher likelihood of complete occlusion, which is biologically plausible given that wider-neck aneurysms may sustain persistent inflow jets and more complex hemodynamics and require a larger surface area to undergo endothelialization for durable exclusion after flow diversion [22–25]. Asymptomatic presentation was also associated with complete occlusion and may reflect earlier detection and treatment before secondary morphologic changes develop (e.g., fusiform or dissecting morphologies); however, this association should be interpreted cautiously because symptom status is likely correlated with aneurysm morphology and other unmeasured factors [26]. In addition, radial access was associated with complete occlusion. Although access route mainly determined by operator technique, catheter systems, case selection, and temporal practice patterns, transradial strategies have been linked to lower access-site complication rates and may provide stable guide support in selected anatomies, potentially facilitating optimal device deployment and wall apposition. [27, 28]

We also observed an association between aspirin ticagrelor therapy and complete occlusion. Because flow-diverter-mediated aneurysm exclusion is primarily driven by flow modification and vessel-wall healing rather than platelet-dependent intra-aneurysmal thrombosis alone [25], this finding may reflect more consistent platelet inhibition in the setting of variable clopidogrel responsiveness, fewer thromboembolic interruptions in therapy, or center-level practice patterns that include platelet-function testing and antiplatelet optimization. [29–31]

Female sex was independently associated with complete occlusion; however, women generally have smaller intracranial arterial diameters in the anterior circulation, including the anterior cerebral artery, which can narrow the technical margin for flow diversion in DACA anatomy by constraining device sizing and increasing the importance of precise wall apposition and luminal preservation [32]. At the same time, smaller parent-vessel caliber could increase the effective mesh density (metal coverage) of a given device and thereby augment the flow-diversion effect, offering a potential mechanistic explanation for the observed association; nevertheless, because vessel caliber, device selection, and procedural strategy were not standardized and residual confounding is likely, this finding should be considered hypothesis-generating. [33, 34]

Finally, device type was not independently associated with complete occlusion, supporting a pragmatic approach to device selection in DACA aneurysms that prioritizes deliverability, size compatibility, operator familiarity, and anatomic considerations over expectations of differential occlusive performance among currently used platforms. Importantly, our study is one of the very few to directly compare FDs specifically in unruptured DACA aneurysms. The absence of significant differences in safety or efficacy among devices suggests that FD selection should be guided primarily by patient anatomy, procedural strategy, and institutional experience rather than assumptions of device superiority. In this context, our findings indicate that clinical and procedural factors—such as aneurysm neck morphology, access route, and antiplatelet regimen—may play a more relevant role in achieving complete aneurysm occlusion than device selection alone. Accordingly, optimization of procedural planning and periprocedural management may be more impactful than the selection of a specific FDs.

This study has several notable strengths. It represents one of the largest multicenter cohorts specifically evaluating unruptured DACA aneurysms treated with FDs, enhancing the generalizability of the findings. However, several limitations should also be acknowledged. First, the retrospective design is inherently subject to selection and reporting biases, with variability in patient selection, procedural techniques, and follow-up practices across centers. Second, some device subgroups were relatively small, limiting statistical power to detect subtle differences. Third, imaging outcomes were assessed locally using different imaging modalities without central adjudication or a core laboratory, which may have introduced inter-center variability despite the use of standardized outcome definitions. Finally, although angiographic follow-up was available for most patients, variability in follow-up duration and shorter long-term follow-up in a subset of cases limited assessment of delayed complications and long-term durability. Future prospective studies with standardized protocols, longer follow-up duration, and evaluation of next-generation surface-modified flow diverters—which are designed to reduce thrombogenicity and may offer a safer profile in distal small-caliber vessels [35]—are needed to validate and extend these findings.

## Conclusion

This multicenter study found that Pipeline, Silk Vista Baby, and FRED Jr. FDs demonstrated comparable safety and efficacy for treating unruptured DACA, with similar rates of complete occlusion, complications, and favorable clinical outcomes. Independent predictors of complete aneurysm occlusion included female sex, asymptomatic presentation, smaller aneurysm neck size, radial access, and the use of aspirin plus ticagrelor therapy. Device type itself was not a significant predictor of occlusion. These findings suggest that device selection should be individualized based on anatomical, procedural, and patient-specific factors rather than presumed differences in device performance.

## Data Availability

Data are available upon reasonable request. Deidentified participant data are available upon reasonable request to the corresponding author.
